# Readiness for Environmental Release of Genetically Engineered (GE) Plants in Uganda

**DOI:** 10.3389/fbioe.2018.00152

**Published:** 2018-10-24

**Authors:** Barbara Mugwanya Zawedde, Musa Kwehangana, Herbert K. Oloka

**Affiliations:** ^1^Uganda Biosciences Information Center, National Agriculture Research Organization, Entebbe, Uganda; ^2^Uganda National Council for Science and Technology, Kampala, Uganda; ^3^Program for Biosafety Systems, Kampala, Uganda

**Keywords:** biosafety framework, biosafety capacity building, GE crops, food safety assessment, risk analysis, risk assessment

## Abstract

Research and development of genetically engineered (GE) crops in Uganda was initiated in 2003 with the launch of a national agricultural biotechnology center at Kawanda in central Uganda. The country has now approved 17 field experiments for GE plants, which were first established in 2006 with the planting of a banana confined field trial that evaluated performance of plants modified to express resistance to black sigatoka disease. Researchers leading the GE experiments have indicated that some of these GE plants are ready for environmental release that is moving beyond confined field testing toward commercialization. The government of Uganda, over the past two decades, has supported processes to put in place an effective national biosafety framework including establishment of a supportive policy environment; creation of a clear institutional framework for handling applications and issuance of permits; building critical capacity for risk analysis; and providing options for public engagement during decision-making. Uganda is ready to make a biosafety decision regarding environmental release of GE plants based on the level of capacity built, progress with priority GE crop research in the country, and the advancement in biosafety systems. Enactment of a national biosafety law that provides for a coordinated framework for implementation by the relevant regulatory agencies will strengthen the system further. In addition, product developers need to submit applications for biosafety approval for environmental release of GE crops so that mechanisms are tested and improved through practice.

## Introduction

Uganda is one of Africa's fasted growing economies. The county's GDP is now estimated at USD 25 billion, up from USD 17 billion in 2012/13 fiscal period. One major driver of growth in Uganda, and indeed many countries in sub-Saharan Africa, has been agriculture. In the 2016/17 fiscal period, agriculture grew by only 1.6%, far below a possible 10% rate that is required to sustain food security in a rapidly growing continent (UBOS, 2017). The slow growth of the agriculture sector is attributable to several biotic and abiotic constraints. Chief among these are pests and diseases in major staple and commercial crops. Drought and related climate change effects continue to limit crop and livestock productivity potential.

The country, through the National Biotechnology and Biosafety Policy 2008, identified biotechnology as a strategic tool to address many crop and livestock production challenges. In the crop sub-sector, biotechnology initiatives were developed to manage pests and diseases that cannot be adequately addressed using conventional breeding techniques. In addition, some crops such as banana require biotechnology approaches as conventional breeding is ineffective in sterile hybrids that form the bulk of the cultivated banana. Genetic engineering has been explored to improve banana and other staple crops such as potato, maize, and cassava.

Crop biotechnology has delivered benefits to millions of farmers in both developed and developing countries. To date, about 18 million farmers cultivate GE maize, soybean, cotton, alfalfa, sugar beet, canola, papaya, potato, and apple among others. Recent evidence shows significant benefits to farmers arising from more efficient production and increased productivity (ISAAA, 2016). Following 22 years of global commercial cultivation of GE crops, Uganda can harness opportunities by adapting and adopting key GE crops such as herbicide tolerant and insect protected crops.

GE crops have been commercialized for more than two decades without demonstrated actual harm to human health and environment (Bawa and Anilakumar, 2013). Potential environmental risks considered include increased weediness and invasiveness, effect on non-target organisms, and changes in the farming system or ecosystem that may impact sustainable conservation of biological diversity. So far, occurrence of these risks has been low after commercialization. This is attributed to the fact that not all GE plants are associated with any or all these risks, a risk assessments conducted by relevant regulatory agencies prior to commercial release of GE crops, and risk management after release, where it is necessary. It is important to note that these potential risks are not only associated with GE plants, however, this paper take a position that fit-for-purpose (“*no more than necessary, and not less than would be harmful to health”*) risk assessment is necessary for any research and development process.

Uganda is steadily developing its regulatory framework to harness the opportunities from modern biotechnology. Compared to its neighboring countries, Uganda's biosafety framework is more advance than in Rwanda, Burundi, Tanzania, Democratic Republic of Congo and Southern Sudan but lagging behind Kenya and Ethiopia. While a number of milestones have been registered and research has progressed to field experiments, the readiness to deploy biotechnology products (environmental release) is yet to be assessed. This paper assesses the country's efforts toward an effective science-based regulatory regime and subsequently the readiness to commercialize GE crops. Focus is on institutional systems and policy environment. Consumer acceptance of modern biotechnology and related market dynamics are not discussed.

## Assessment of the current biosafety policy environment and practices

A biosafety regulatory framework is necessary to ensure that human health and the environment are protected from possible adverse effects of products of modern biotechnology. The biosafety system also provides a basis for public confidence and for legal certainty for research organizations and private sector (industry). The major components of a functional national biosafety framework (NBF) include: a supportive policy environment; an institutional framework for handling applications and issuance of permits; a system for risk analysis and decision making; and a mechanism for public participation in biosafety decision-making. The government of Uganda, over the past two decades, has supported the processes to enable development of these key elements.

### Policy environment

Uganda actively participated in the negotiation and subsequently ratified the Cartagena protocol on Biosafety in 2001. The country took further steps to provide for the obligations of the Cartagena protocol. An interim biosafety system to regulate modern biotechnology research and development has been adopted in the absence of holistic legislation. Uganda National Council for Science and Technology (UNCST) was designated the Competent National Authority that provides regulatory oversight for GE research and development initiatives. The UNCST Act, 1990 gives it mandate to clear all scientific research and development activities in the country.

As part of efforts to develop a holistic biotechnology and biosafety regulatory and development framework, Uganda adopted the National Biotechnology and Biosafety Policy in 2008. The Policy recognizes GE as a tool that can be used to enhance agricultural productivity, improve food and nutrition security, promote conservation and sustainable use of natural resources, and enhance human and environmental health. The Policy, under Section 5.4 commits the Government of Uganda to develop legislative instruments to regulate modern biotechnology applications.

### Institutional framework

**Figure 1:**
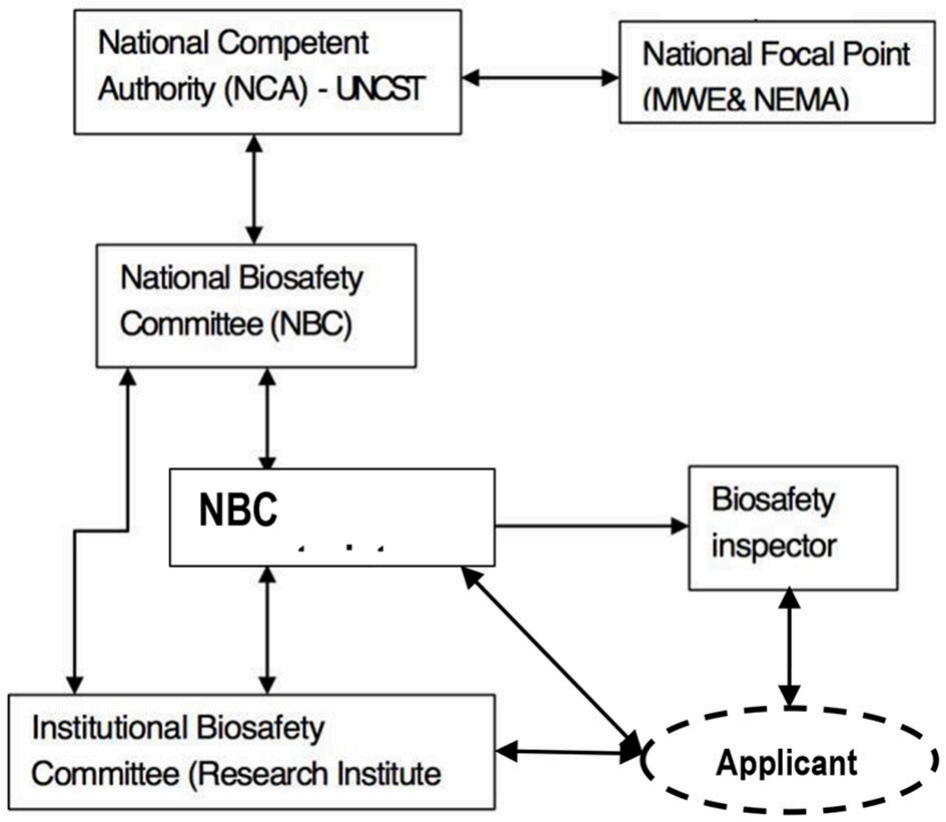
Schematic diagram of the Uganda's biosafety institutional framework.

The National Biotechnology and Biosafety Policy (2008) requires establishment of an institutional framework to support the regulatory process and articulate strategies for capacity building, infrastructural development and technology transfer. Uganda has established an interim institutional framework to operationalize the biosafety regulatory system. The current institutional biosafety framework, as described below, comprises of the national competent authority, the national focal point, the national biosafety committee, the inspection mechanism and institutional biosafety committees (Figure 1).

The UNCST is the designated national competent authority to supervise and coordinate implementation of biosafety in the country. The competent authority houses the secretariat of the national biosafety committee. Among its functions, the competent authority approves the development, testing and use of GE products in Uganda, ensures safety of biotechnology to human health and environment during development and testing of GE products and also updates and informs the National Focal Point on matters related to biosafety and biotechnology.

In 1996, the UNCST established the national biosafety committee (NBC) as its technical advisory body for matters concerning biosafety. The main function of the committee is to provide technical advice on biosafety issues to the government particularly with respect to the assessment of benefits and risks associated with modern biotechnology applications and processes. The NBC comprises of relevant experts with competence to review and evaluate risks and benefits of biotechnology research and development activities. The current NBC consists of the following expertise: human health, animal health, plant or animal conservation / biodiversity, biotechnology, social science, agricultural regulation, entomology, legal, environmental chemistry, trade, standard, agriculture, and consumer rights. The NBC can draw upon more experts when necessary.

Institutional biosafety committees (IBCs) have been established in some agencies engaged in biotechnology research and development. IBCs provide a linkage between the NBC and researchers. IBCs are responsible for the initial in-house quality assurance by approving, monitoring, and reviewing contained experiments and recommending confined experiments to the NBC. IBCs also ensure that research by the applicant is done in accordance with conditions of approval set by the NBC. The most active IBC was established by the National Agricultural Research Organization (NARO) in 2004. This IBC has reviewed and overseen more than 20 GE research activities at contained and confined levels.

The Cartagena Protocol on Biosafety requires parties to establish National Focal Points to liaise with the Convention on Biological Diversity (CBD) Secretariat on matters regarding the implementation of the Protocol. The government of Uganda designated the Ministry of Water and Environment as the National Focal Point (NFP) for the Cartagena Protocol on Biosafety. The Competent Authority works closely with the National Focal Point.

UNCST with technical support from development partners like Program for Biosafety Systems (PBS), African Biosafety Network of Expertise (ABNE), and International Center for Genetic Engineering and Biotechnology (ICGEB) has built inspection capacity to oversee and/or enforce regulatory compliance to the terms and conditions of approval. Inspectors were identified from UNCST, the Ministry of Agriculture, Animal Industry and Fisheries (MAAIF), NARO, Uganda National Bureau of Standards (UNBS), universities, Ministry of Water and Environment and the National Environment Management Authority (NEMA). Trained and certified inspectors are designated by UNCST and deployed whenever required.

### Capacity for risk analysis

Uganda has made tremendous progress in developing human and infrastructural capacity for risk analysis, and biosafety management and enforcement (UNCST, 2016). Currently, there are nine universities that offer biotechnology related courses within a wide scope of other biology-based disciplines. Makerere University, Kyambogo University, Uganda Christian University, and Bugema University in Central region, Busitema University, and Islamic University in Eastern Region, Gulu University in Northern region, and Bishop Stuart University and Mbarara University in Western region. Uganda has also strengthened its biosafety system through short-term training programs for its biosafety practitioners including NBC and IBC members and inspectors.

The country has built more than 10 public biotechnology laboratories, hosted at various universities and research centers. These facilities are capable of conducting basic and advanced biotechnological applications including molecular screening, bioinformatics, plant transformation, tissue culture, and nutrition assays among others. NARO has the most advanced among facilities hosted at Kawanda and Namulonge. About six private agricultural biotechnology institutions are operational, specializing in micro-propagation of coffee, banana, sweet potato, pineapple and potato. There are currently two regulatory focused laboratories addressing GE food safety and GE testing. The existing human and infrastructural capacity can readily be drawn upon for risk analysis, enforcement and management.

### Status of GE research and development in Uganda

The first application for research using genetic engineering was made in 1992 when Makerere University requested for approval to test bovine somatotropin hormone developed using recombinant DNA technology. Due to limited biosafety capacity at the time, the application was not approved. Plant genetic engineering research in Uganda effectively started in 2003 after H.E. the President of Uganda launched the National Agricultural Biotechnology Center in NARO-Kawanda, central Uganda. Field experiments testing GE crops were initiated 11 years ago with the planting of the first banana confined field trial that evaluated performance of plants genetically engineered to express resistance to black sigatoka disease. The country has now approved 17 field experiments involving GE crops addressing specific production or nutrition challenges (Table 1). The GE crops under testing have been improved for various traits and are at different stages of evaluation.

**Table 1:** Status of GE crop field research in Uganda.

**Crop**	**Trait**	**Developer/partners**	**Period of Research**	**Regulatory status**	**Estimated commercial release timeline**
Banana	Bacterial wilt resistance	NARO; AATF	2010 to date	Multi-location CFTs	2021
	Resistance to black sigatoka	NARO	2006–2009	CFT	–
	Resistance to nematodes	NARO	2012 to date	CFT	2021
	Pro-Vitamin A enhancement	NARO, QUT	2011 to date	CFT	2019
Maize	Drought tolerance	NARO, AATF	2010–2014	CFT	2018
	Insect pest resistance	NARO, AATF	2012–2013	CFT	2018
	Drought tolerance and insect resistance stack	NARO, AATF	2015 to date	Multi-location CFTs	2018
Cassava	Brown streak disease resistance	NARO, Donald Danforth Plant Science Center (DDPSC)	2010 to date	Multi-location CFTs	2019
	Cassava mosaic disease resistance	NARO, DDPSC	2011–2012	CFT	–
Potato	Late blight resistance	NARO, CIP	2015 to date	Multi-location CFTs	2019
Sweet potato	Resistance to viral diseases	NARO	2013–2014	CFT	–
Rice	Nutrient and water efficiency	AATF, NARO	2012 to date	CFT	–
Soybean	Herbicide tolerance	NARO	2016 to date	Contained research	2019
Cotton	Herbicide tolerance and insect resistance	NARO	2007–2012	CFT	–

*Source: Research scientists. QUT, Queensland University of Technology; AATF, African Agricultural Technology Foundation; CIP, International Potato Centre*.

#### Banana

Currently NARO is conducting three confined field trials (CFTs) for GE banana at the National Agricultural Research Laboratories (NARL) in Kawanda. Vitamin A rich banana is the most advanced trial as it approaches advanced food safety and nutritional studies. Bacterial wilt resistant banana has been approved for multi-location field testing in two additional sites; south-Western Uganda in Mbarara and Western Uganda in Hoima. Trials are also underway for weevil and nematode resistance at Kawanda.

#### Cassava

Trials have been conducted for GE virus resistant cassava from 2009. While the first trials focused on resistance to cassava mosaic disease, ongoing regulatory trials in Mubuku, Kasese aim at resistance to brown streak disease. Cassava brown streak disease has now become one the greatest challenges to cassava production in the country, affecting nearly all districts where the crop is cultivated. Annual yield losses are estimated at more than USD 40 million. Progression to regulatory trials under the Virus Resistant Cassava for Africa (VIRCA) project implies the technology has proved effect under field evaluation.

#### Maize

Uganda experiences occasional moderate to severe drought in selected regions. In the 2016 to 2017 cropping season, severe drought caused significant food insecurity for many families. Drought tolerant maize developed using genetic engineering has been tested for more than 8 years in the country. Trials were initially conducted at Mubuku, Kasese but with the inclusion of stem borer resistance—also developed using GE techniques, other trials were setup in Namulonge, at the National Crops Resources Research Institute (NaCRRI). These research efforts are part of the Water Efficient Maize for Africa (WEMA) Project and build on proven technologies commercialized in other countries. In Uganda, the WEMA trials are among the most advanced toward environmental or general release.

#### Potato

NARO scientists are evaluating GE potato for resistance to late blight disease at three locations in Uganda. Experiments are underway in south Western Uganda (Kabale), Western Uganda (Kabarole), and Eastern Uganda (Bulambuli). Late blight of potato continues to be a major worldwide threat to potato production and management has been largely through application of fungicides. Integration of late blight resistance into the potato will offer farmers greater flexibility and efficiency in managing this disease.

#### Soybean

Makerere University, through the College of Agricultural and Environmental Sciences, is currently conducting contained testing of herbicide tolerant soybean. The trials were approved for introgression of proven Roundup Ready technology into locally adapted varieties. Field evaluations will be conducted once stable segregants are identified.

NARO scientists have indicated that many of these research efforts are awaiting an enabling policy environment to move toward environmental release and commercialization. Some technologies are already proven effective in other countries including *Bt* maize and Roundup Ready soybean that have been commercially cultivated for many years. The current experiments in Uganda, including cross-breeding experiments involving proven technologies, imply the need to understand the country's readiness for environmental release of GE crops.

## Appraisal of the essential requirements of decision making for environmental release of GE plants

The process of decision-making regarding environmental release of GE plants is different from that of contained and confined research. While Uganda was able to conduct confined field tests under the provisions of the UNCST Act that governs all STI research, the country took a policy decision that environmental release and commercialization of GE organisms should be guided by an explicit legislative instrument. Biosafety legislation will guide the institutional mechanisms and biosafety decision making systems.

### Regulatory policy

Proposed biosafety legislation was approved by the country's Cabinet in 2012 as the National Biotechnology and Biosafety Bill. The bill provides the scope of regulatory coverage; establishment, description and functions of the decision-making authorities; processes and timelines for different approvals; provisions for conducting risk assessment; socio-economic considerations; monitoring for compliance; and public participation. It also provides for other administrative structures including handling confidential information; enforcement; appeal; fees; labeling; and liability and redress. This proposed legislation was first presented in Parliament in 2013 and was later approved for passage as the National Biosafety Act, 2017. Assent to this law was deferred and the bill is under revision in Uganda's Parliament.

It is important to note that biosafety legislation is not implemented in isolation. Additional considerations for environmental release of GE plants may be provided in other national legislations including: The National Environmental Act (Cap 153); the Plant Protection and Health Act (Cap 31); the Seed and Plant Act (2007); and the Plant Variety Protection Act (2014) (Zawedde et al., 2012).

Uganda has also signed a number of international treaties that may be considered during decision making for environmental release of a GE crop. The Cartagena Protocol on Biosafety (CPB, 2000) that requires Uganda, as a Party, to provide for *adequate level of protection for safe transfer, handling, and use of living modified organisms that may have an adverse effect on the conservation and sustainable use of biological diversity, taking into account risks to human health and specifically focusing on transboundary movements*. Uganda under the proposed legislation has adopted the risk assessment guidelines under CPB in the proposed biosafety legislation. The Codex Alimentarius provides a *collection of internationally recognized standards, codes of practice, and guidelines relating to food safety*. Uganda National Bureau of Standards (UNBS) adopted Codex guidelines to develop data interpretation guidelines for use during food safety assessment for GE crops.

Organization for Economic Co-operation and Development (OECD) has *Consensus Documents* that the country may consider to provide *science-based information during environmental risk assessment*. The World Trade Organization treaties including General Agreement on Tariffs & Trade (GATT, 1994), Agreement on the Application of Sanitary and Phytosanitary Measures (SPS Agreement) and the Agreement on Technical Barriers to Trade (TBT Agreement) require Uganda as a Member State to take actions to prevent potential barriers to trade including regulating biotechnology through the adoption of biosafety measures. Decision-making may also be affected by the on-going efforts by the African Union, the Common Market for Eastern and Southern Africa (COMESA), and the East African Commission to harmonize regulation of biotechnology and its products.

For the environmental release of GE crops, Uganda can be guided by implementing provisions in relevant existing national legislation such as the National Environment Act and the Seed and Plant Act while complying with relevant requirements under regional and international obligations. However, this readiness will be greatly enhanced by enactment of an explicit biosafety law that would provide a more coordinated regulatory framework for GE organisms.

### Proposed institutional framework for biosafety regulation in Uganda

**Figure 2:**
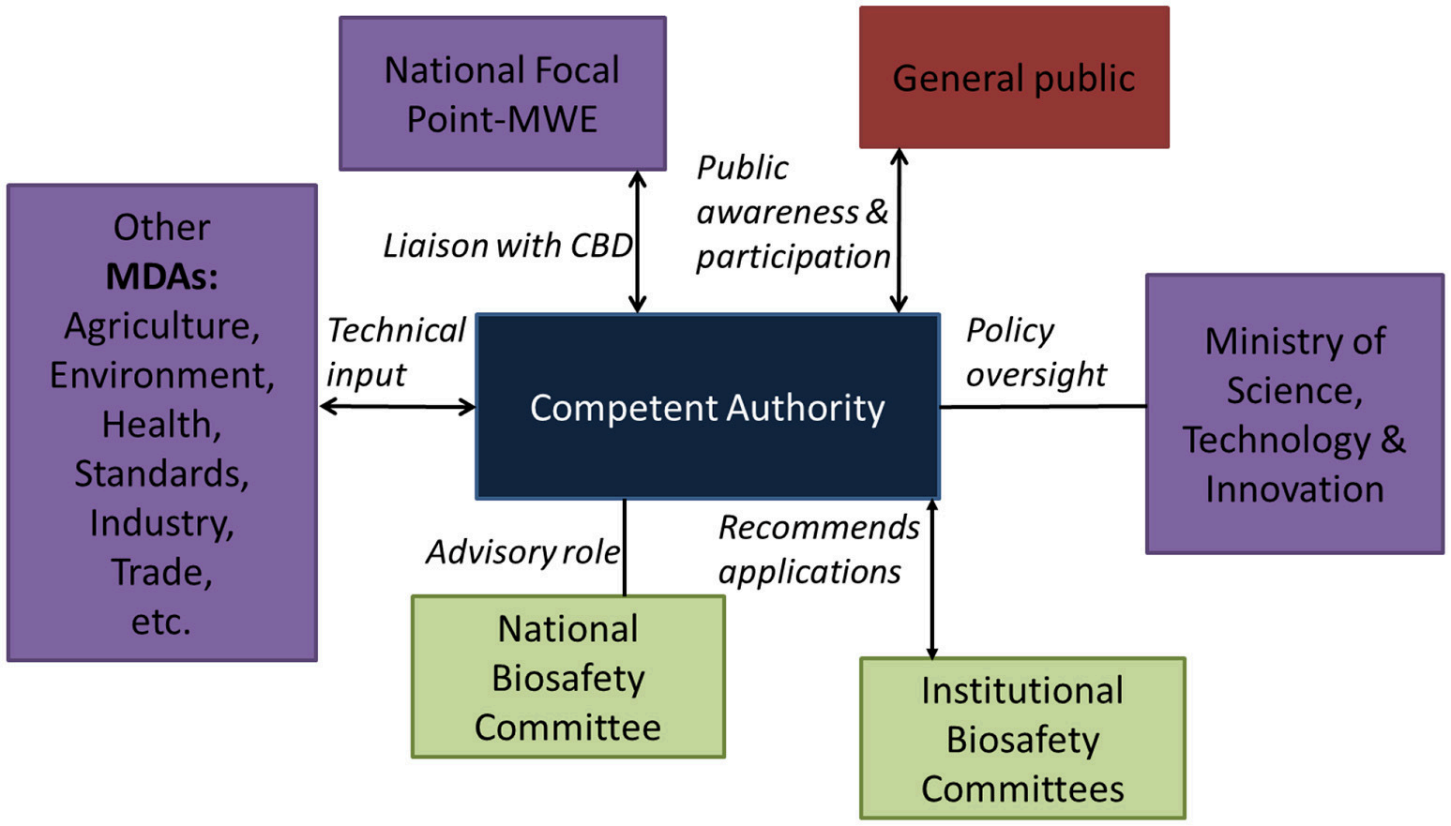
Proposed institutional framework (Under the Bill for National Biosafety Act, 2017).

A clear institutional framework has been proposed in the new legislation (Figure 2). This framework aims to support sound decision-making while building a trusted regulatory system that demonstrates competence, credibility and integrity.

The proposed role of the *Competent Authority* is to link all actors together to ensure safe application of modern biotechnology. The ministry responsible for science and technology will play a policy oversight role as well as act as the national focal point for the Cartagena Protocol for Biosafety. The national focal point role was previously the responsibility of environment ministry. Other relevant ministries, departments and agencies are expected to continue respective mandates of relevance to environmental release of GE crops. The National Environmental Management Authority (NEMA), which is the principal agency in Uganda for the management of the environment is mandated to coordinate, monitor and supervise all activities in the field of the environment. As such, NEMA will play a significant role of participating in the pre-release environmental risk assessment, and in closely monitoring the possible post-release adverse effects of GE plants on conservation and sustainable use of biodiversity. The Government of Uganda is currently at advanced stages of amending the National Environment Act (1995) to among other considerations, codify environmental risk assessment of GE organisms prior to general release.

In addition to the current role of overseeing inspection of research for compliance with phytosanitary measures, the Ministry of Agriculture, Animal Industry and Fisheries through its Crop Protection department will play a role of regulating import and export of GE organism and regulated agricultural products. Upon approval for environmental release of a GE crop by the competent authority, the agriculture ministry will ensure that variety release procedures are followed prior to commercial release of a GE crop. The Crop Protection department may be delegated by the competent authority to participate in post-release monitoring of the GE plant.

The *Ministry of Health* through its National Drug Authority (NDA) is responsible ensuring the availability of efficacious and cost-effective drugs to the entire population of Uganda. A number of drugs are generated from plants. Plans are underway to use genetic engineering to enhance production of drugs active ingredients in local herbs. It is expected that if some of these trials prove promising and safe, then NDA will play a critical regulatory and safety assessment role prior to approval of the drugs for wider use and application in Uganda. This makes the regulatory agency for drugs in Uganda an important stakeholder in biosafety management.

*Ministry of Trade, Industry and Co-operatives* is an important player in environmental release of GE plants because it has to advise on socio-economic considerations such as effects on industrial development and on trade. This ministry also provides policy oversight on the *Uganda National Bureau of Standards (UNBS)*. UNBS enforces standards in protection of public health and safety and the environment against dangerous and sub-standard products. The main relevance of UNBS for biosafety is their role in ensuring standards for safety of foods (both locally produced and imported) before they are allowed to be sold or distributed on the Ugandan market.

The proposed institutional framework is inclusive. Its efficiency for environmental release of GE plants will benefit from strengthening the linkages and working relations among relevant ministries, departments and agencies; defining a clear mechanism for compliance enforcement, providing feasible mechanisms for public participation, and building the relevant capacity for risk assessment and risk management within the relevant regulatory agencies.

### Capacity for risk assessment and risk management

Designing and implementing a fit for purpose risk assessment is pertinent for effective risk avoidance, reduction or management. Readiness for environmental release of GE plants requires strengthening necessary capacities for environmental risk assessment and food safety assessment.

#### Environmental risk assessment (ERA)

Environmental risk assessment (ERA) is necessary prior to environmental release of GE plants (Macdonald, 2017). ERA is conducted on a case-by-case basis depending on the GE traits or host plant and the receiving environment. It considers potential risks such as increased weediness and invasiveness, effect on non-target organisms, and changes in the farming system or ecosystem that may impact sustainable conservation of biological diversity. The ERA process involves problem formulation, hazard and exposure evaluation, and risk characterization (Layton et al., 2015). The key factors considered during the ERA process include the host crop that has been improved, the introduced trait and the receiving environment.

Problem formulation involves clear identification of policy requirements and relevant protection goals. Considerations are then made based on existing data and conceptual models to identify biodiversity likely to be exposed, potential harm and exposure pathways (Wolt et al., 2010). Once protection goals and exposure pathways are identified, resources can then be focused on generating missing data necessary for decision making on acceptability of the risk. Hazard and exposure evaluation is typically based on a tiered approach that commonly uses surrogate species and different exposure levels to increase the efficiency of data collection that may be necessary for ERA (Romeis et al., 2006). A tiered approach is often applied in understanding risks to non-target organisms as identified during the problem formulation stage. During risk characterization, available data is utilized to determine the potential consequences under real environmental conditions. Following the outcomes of the ERA, risk management options will be considered where necessary to mitigate or reduce the level of risks to protect human health and the environment. Typically contingency plans for risk management may be required as part of any conditions imposed during authorization for environmental release.

ERA requires having expertise in key relevant fields such as environmental quality; environmental chemistry; ecotoxicology; environmental risk assessment; microbiology; biochemistry; and handling, monitoring, and remediation of pollution (Soares, 2015). Competences for risk management will stretch beyond technical knowledge to good understanding of procedural aspects of policy making and inspection, and communication with stakeholders. Effective implementation of risk analysis for environmental release of a GE plant will require having a small group of well trained and skilled regulators (Macdonald, 2017).

At national level, most of the required ERA expertise already exist within a number of regulatory institutions, research agencies, universities, and private sector in Uganda. The expertise can be drawn upon to contribute to the risk analysis process necessary for environmental release. At least three scientists working within research and regulatory institutions have received advanced training in ERA, while over 60 scientists and regulators have attended short courses on biosafety risk assessment since 2004.

Uganda's readiness for environmental release of GE plants will require Government investing in strengthening the human capacity within the regulatory agencies by training more risk assessors and risk managers. This may be achieved by conducting tailored, hand-on training programs to strengthen the existing skills, collaborating with experienced risk assessors, and/or through Masters training programs (Komen and Koch, 2017). Strengthening biodiversity conservation capacities will also be necessary.

#### Food safety assessment

GE plants are typically are subjected to food and feed safety assessment that consider potential risks of increased expression of toxic or allergenic compounds, and changes in the nutritional value. In view of the potential impact of biotechnology on the food industrial sector and current research efforts in staple crops, there is a clear need for Uganda to take initiatives to build autonomous capability in food safety assessment and management. Local capability in food safety will be required in selected regulatory agencies such as UNBS, the Government Analytical Laboratory, and food science laboratories in public research and tertiary institutions.

In the recent past, the Program for Biosafety Systems (PBS), African Biosafety Network of Experts (ABNE), and International Center for Genetic Engineering and Biotechnology (ICGEB) have supported building skills of regulators (Table 2). The training sessions have focused on building skills to review dossiers for environmental release of GE plants, interpretation of the risk assessment data, and they have worked with some of the relevant agencies to develop standard operating procedures necessary for GE research.

**Table 2:** Scientists trained on basic risk assessment for environmental release.

**Agency**	**No. of trained personnel**	**Existing biosafety competence**	**Biosafety role**
UNCST (includes NBC)	17	Risk assessment; Biosafety reviews; development of guidelines and SOPs	Competent Authority
MAAIF	8	Inspection; risk assessment	Inspections; biosafety support
UNBS	5	Food/feed safety assessment; risk assessment; inspections; development of guidelines	Food safety support
NEMA	5	Inspection; risk assessment	Biosafety support
NARO	15	Risk assessment; dossier preparation; compliance management	Product development; biosafety support (thru IBC)
Makerere University	13	Risk assessment; Inspections	Biosafety support; Training

Adapted with modification from Baguma et al. (2013); personal communications and consists of estimates

Uganda's readiness will be influenced by willingness to use, and confidence, by our regulatory system in data obtained through outsourcing safety assessments. In such cases, we will need to develop capacity for data transportability and interpretation. Data transportability is the application of data produced in one geographic location to support the safety assessment of that same product in another location (Delaney, 2010). Data generated elsewhere, particularly on food and feed safety, is expected to be useful for similar assessments in Uganda. Data transportability helps to overcome the challenge of allocating resources to carry out comprehensive analytical requirements to establish the “identity” or safety of the proteins in the products. The Uganda National Bureau of Standards has already developed guidelines for data transportability for food safety assessment.

#### Infrastructural capacity

Uganda has also progressively built a critical infrastructural capacity (Table 3), which can be used to conduct ERA and risk management. This was achieved in collaboration with development partners like USAID, Gates Foundation, Howard Buffet, DFID, Rockefeller, FAO as well as with regional initiatives such as ASARECA, BIOEARN, Biosafe Train, among others. Due to the constant and rapid evolution of this science, the necessary level of infrastructural capacity will always be a “moving target” (OECD, 2009). Our readiness will also benefit from Government providing an enabling environment to increase private sector investment in laboratories that can conduct such assessments.

**Table 3:** Laboratory and field infrastructure for GE Plant testing.

**Service**	**Availability in labs and facilities and institutions**	**Comments**
Molecular characterization	NARL, NaCRRI, Government Analytical lab	Staff trained but more equipment needed
Compositional analysis	UNBS, NaCRRI	Mock tests are underway
Full food and feed safety assessment	UNBS (microbial and toxicity studies)	Staff trained in data transportability; mock tests needed
GMO testing	MAAIF, NaCRRI, NARL	Staff trained and equipment available

### Public awareness and participation

Release of GE plants into the environment is of interest to a wide spectrum of the community, including farmers and their associates, government agencies, non-government, and civil society organizations, grassroots communities, media, academia and private sector. Therefore, public awareness is an integral component of every step in regulatory decision-making. Public participation is also critical in the regulatory process for environmental release of GE plants because it allows decision-making to be based on up-to-date and relevant scientific information, and socio-economic considerations for the receiving environment and community (Keese, 2013).

Public awareness efforts to support establishment of a National Biosafety Framework have been on-going since 1996. UNCST, UNEP-GEF, PBS, Uganda Biotechnology and Biosafety Consortium (UBBC), Uganda Biosciences Information Center (UBIC), Science Foundation for Livelihoods and Development (SCIFODE), Open Forum on Agricultural Biotechnology in Africa (OFAB-Uganda Chapter), Tropical Institute of Development Innovations (TRIDI), Cornell Alliance for Science, ISAAA Afri-Center, ABNE and NARO biotech-research projects such as Water Efficient Maize for Africa (WEMA), Virus Resistant Cassava for Africa (VIRCA-Plus), Banana 21 and Banana Bacterial Wilt resistance project have been key players in enhancing public awareness. These efforts have also been focused on enhancing public confidence in the biosafety regulatory system.

In the last 5 years, there has been a lot more public engagement on biosafety focusing on discourse related to the proposed legislation. Trainings were also conducted to empower voices within various stakeholders' groups to support putting in place a functional biosafety system in Uganda. The awareness activities targeted influential champions in various stakeholders' groups including politicians, policy makers, scientists, regulators, media practitioners, extension agents, youth and women groups, and community, opinion, religious, cultural and farmers' group leaders.

**Figure 3:**
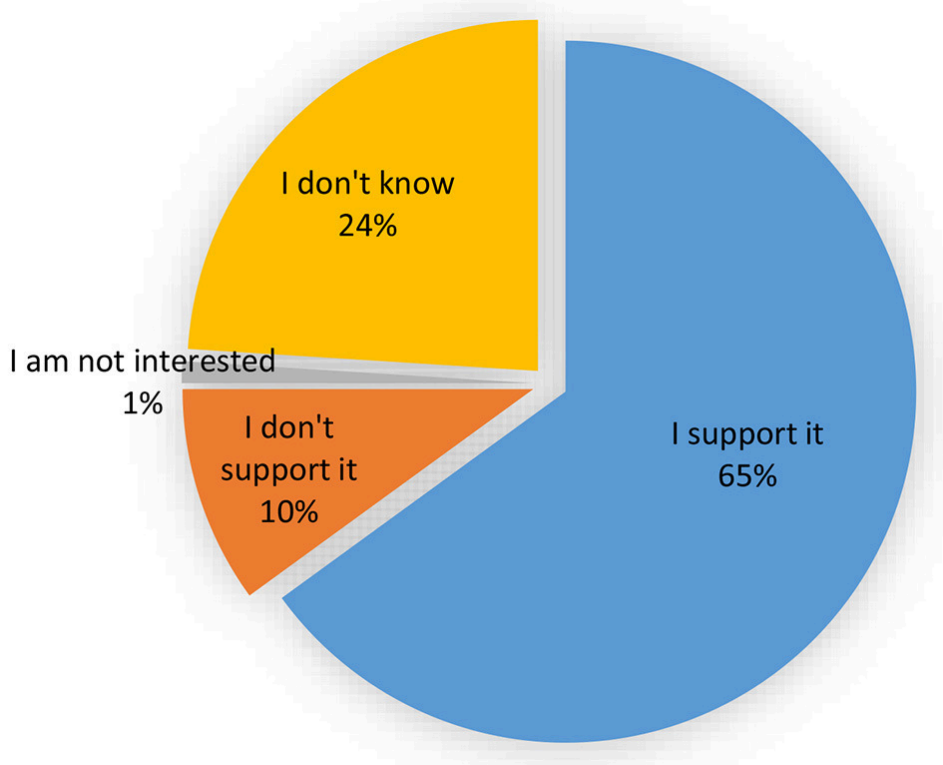
Viewpoints of respondents toward having in place a biosafety system to regulate modern biotechnology.

A recent study conducted by UBIC to assess public knowledge, attitude and perception toward modern biotechnology regulation showed that 65% of the respondents supported having in place a functional biosafety system (Figure 3). The study was conducted in 12 districts distributed in all the four regions of the country, and 653 respondents representing various stakeholders participated.

The study further showed that 10% of the respondents did not support having a having a biosafety system because they believed it was synonymous with introduction of GE crops that they oppose. This group together with the respondents (24%) who did not know whether we need a biosafety system are a clear indication of the need for more engagement of key stakeholders relevant to establishment of a functional biosafety system. It is also anticipated that as the country progresses in modern biotechnology application, critics will boost anti-biotech campaigns that may reduce public trust in the biosafety regulatory system. A biosafety communication strategy has been developed by the Ministry for Science, Technology and Innovation to increase public appreciation and confidence in the biosafety system. There is therefore a need to mobilize resources and to strengthen the existing partnerships so that more influential spokespersons are empowered and engaged to support an efficient biosafety regulatory system.

## Challenges associated with mainstreaming biosafety in Uganda

The government of Uganda has made efforts to mainstream biosafety management, policy development and education through national policies such as Vision 2040 and the National Development Plan II. Further integration of biosafety in regulatory and research agencies is constrained by a number of factors. Existing laws are not explicit on biosafety or regulation of GE techniques and products and this can cause conflicting mandates in different regulatory institutions. Most of the existing laws and policies were formulated before Uganda ratified the Cartagena Protocol on Biosafety. Delays in the passage of the national biosafety law is a major set-back in mainstreaming biosafety across sectors.

Another major limitation to mainstreaming biosafety in national systems is the lack of appreciation of the role of an effective biosafety framework in supporting safe advancement of biotechnology applications. This has affected capacity building in various regulatory institutions and research centers. At present, only two institutions have expressed interest in having an institutional biosafety committee. Biosafety awareness building efforts need to focus on key regulatory and research agencies and private sector. Capacity is needed in these agencies for key biosafety areas such as risk assessment and management, GE screening and identification, addressing socio-economic issues, and risk communication.

The high turnover of regulators within key agencies also affects the country's efforts to build an effective biosafety system. While overall capacity exists within the country to regulate many aspects of biosafety noted above, new regulators always require refresher training to understand the issues, best practices, and regulatory procedures. This can be addressed by staggering the appointment of new regulators into NBC, and IBC.

Activism against biotechnology advancement by selected groups in the country has further constrained the mainstreaming of biosafety in national institutions, including the enactment of a biosafety law. As the benefit of a biosafety system is not clearly understood by many leaders, the subject is often associated with advancement of genetically modified organisms (GMOs) that is a divisive subject matter in many developing countries yet the purpose of a biosafety system is regulation.

## Prospects for advancement of GE technology in Uganda

Uganda recognized the value of genetic engineering in the late 1990s when it developed a comprehensive poverty eradication plan (PEAP) and plan for modernization of agriculture (PMA) that supported research into and evaluation of GE technologies to address crop production challenges. The country continues to show high level policy interest and action for integration of science and technology in national development. In 2016, the country created a fully-fledged ministry for science, technology and innovation to guide and support advancement of science. The government also established an innovation fund—capitalized initially with about USD 10 million for the first year—to support scientific research and development activities. The new science ministry has been instrumental in leading efforts toward an evidence-based biosafety framework in Uganda.

New initiatives using GE tools under consideration in Uganda may positively influence the ability to make decisions on GE plants. The country, is considering GE mosquito research to address the malaria burden, that costs the country more than USD 100 million to manage each year. National scientists have also collaborated with international private sector partners to test and produce anti-tick vaccines developed through GE technology. The commitment of policy leaders to develop a bio-economy strategy will aid in harnessing some of these tools and products.

The existence of capacity for GE research and capacity for regulation as evidenced by the high numbers of regulators and scientists trained and participating in various aspect of regulation gives greater confidence to stakeholders on the readiness of the country's systems for environmental release of GE plants. Lawmakers had in the past raised issues about the capacity to regulate. Capacity development is nonetheless a continuous effort. Regulatory capacity in Uganda has largely developed in tandem with research progress. This implies that steps have to be taken toward environmental release for the country to build the necessary experience for effective regulation. GE crops approved elsewhere have been proven to be safe using appropriate risk assessment systems. These will form a clear guide for countries such as Uganda where hitherto unreleased GE plants and trait combinations–such as bacterial wilt resistant banana—are being considered.

Opportunities exist to support additional capacity development as may be necessary. A number of national and international initiatives exist that can contribute to these efforts. Some of these initiatives include: African Biosafety Network of Expertise of the New Partnership for Africa's Development (NEPAD); Program for Biosafety Systems (PBS); International Centre for Genetic Engineering and Biotechnology (ICGEB); the International Plant Biotechnology Outreach program of the University of Ghent; Uganda Biotechnology and Biosafety Consortium (UBBC) and Uganda Bioscience Information Centre (UBIC).

Relevant GE crops for Uganda's agriculture have been approved for environmental release in neighboring countries such as GE cotton in Kenya and Ethiopia. This increases the likely for GE crops going through cross border trade and seed exchange. Regional advancements in Ethiopia, Kenya, Malawi and Tanzania toward environmental release of GE crops will build further confidence among Ugandan stakeholders, regulators and policy leaders. As a member of various regional markets such as the East African Community and the Common Market for Eastern and Southern Africa (COMESA), there is opportunity in exploring biotechnology solutions given then improving policy environment within the regional blocks.

## Actionable recommendations and conclusion

This review proposes some actionable recommendations for consideration by the relevant ministry and competent authority. The ministry responsible for biosafety needs to strengthen and build new strategic partnerships to support enactment of a national biosafety law and related instruments such as regulation and guidelines. An effective biosafety regulatory system will necessitate the participation and cooperation of other regulatory agencies involved in environmental management, standards, food safety and plant protection, among others. Operationalization of the law will provide a more coordinated regulatory framework with clear role and responsibilities that will contribute to strengthening the linkages among relevant ministries, departments and agencies.

The competent authority will need to identify, appoint and empower a small group of well-trained and skilled regulators to constitute the NBC. There will be need to develop and maintain a roster of experts that the regulators may call upon to contribute to the risk analysis process. Some of the areas of expertise to be considered include biochemistry; bioremediation; environmental quality; environmental chemistry; ecotoxicology; environmental risk assessment; food science; food safety; microbiology; molecular biology; regulatory enforcement; science communication; science policy among other.

The ministry should also support working relations among the relevant agencies by prescribing mechanisms for good information flow and facilitating periodic networking opportunities. Among these agencies that include NEMA, UNBS, and Ministry of Agriculture, there will be need for capacity building to delineate biosafety considerations from other mandated regulatory considerations of these institutions. Government will need to invest in training more risk assessors and risk managers within these agencies. This may be achieved by conducting short-term to long-term training programs and exchange visits.

The ministry should engage Government to enhance its strategies for attracting science, technology and innovation investment by private sector. An enabling environment together with increased demand from scientists will to increase private sector investment in laboratories that can conduct such assessments.

In some case, outsourcing risk/safety assessment will be the better option. To prepare for such cases that are likely to increasingly become common, the country needs to develop capacity for data transportability and interpretation.

Enhancing awareness, and building confidence, among key stakeholders will require strengthening existing, and building new, partnerships to implement the biosafety communication strategy developed the ministry. Effective communication channels identified by the recent UBIC study will be used to deliver targeted messages to the different stakeholders' groups.

This review also indicates significant progress toward development of key systems necessary for environmental or general release of GE plants. Clear capacity exists for risk assessment. Institutional structures to support approval already exist and will be strengthened by explicit legislation once passed.

As with all GE plants approvals worldwide, a case-by-case consideration will be made by the relevant regulatory system. It is our opinion that Uganda is ready to make a biosafety regulatory decision for environmental release of GE plants based on the level of capacity built, progress with priority GE crops research in the country and advancement in biosafety system.

Enactment of a national biosafety law that provides for a coordinated framework for implementation by the relevant regulatory agencies will strengthen the system further. In addition, product developers need to submit applications for biosafety approval for environmental release of GE crops so that mechanisms are tested and improved through practice.

## Author contributions

BZ, identified the essential requirements of decision making and assessed the current status to determine what is needed to enhance the readiness of Uganda for environmental release of GE plants. MK, provided update on the current status of the biosafety and identified challenges associated with mainstreaming biosafety in Uganda. HO, prepared the introduction and provided an update on the current status of GE crop research and development and identified prospects for advancement of GE technology in Uganda.

### Conflict of interest statement

The authors declare that the research was conducted in the absence of any commercial or financial relationships that could be construed as a potential conflict of interest.
